# MicroRNAs and angiogenesis: a new era for the management of colorectal cancer

**DOI:** 10.1186/s12935-021-01920-0

**Published:** 2021-04-17

**Authors:** Yufei Tang, Shaoqi Zong, Hailun Zeng, Xiaofeng Ruan, Liting Yao, Susu Han, Fenggang Hou

**Affiliations:** 1 Shanghai Municipal Hospital of Traditional Chinese Medicine, Shanghai University of Traditional Chinese Medicine, Shanghai, 200071 China; 2 Graduate School of Shanghai, University of Traditional Chinese Medicine, Shanghai, China

**Keywords:** MiRNA, Colorectal cancer, Angiogenesis

## Abstract

MicroRNAs (miRNAs) are a class of small noncoding RNA molecules containing only 20–22 nucleotides. MiRNAs play a role in gene silencing and translation suppression by targeting and binding to mRNA. Proper control of miRNA expression is very important for maintaining a normal physiological environment because miRNAs can affect most cellular pathways, including cell cycle checkpoint, cell proliferation, and apoptosis pathways, and have a wide range of target genes. With these properties, miRNAs can modulate multiple signalling pathways involved in cancer development, such as cell proliferation, apoptosis, and migration pathways. MiRNAs that activate or inhibit the molecular pathway related to tumour angiogenesis are common topics of research. Angiogenesis promotes tumorigenesis and metastasis by providing oxygen and diffusible nutrients and releasing proangiogenic factors and is one of the hallmarks of tumour progression. CRC is one of the most common tumours, and metastasis has always been a difficult issue in its treatment. Although comprehensive treatments, such as surgery, radiotherapy, chemotherapy, and targeted therapy, have prolonged the survival of CRC patients, the overall response is not optimistic. Therefore, there is an urgent need to find new therapeutic targets to improve CRC treatment. In a series of recent reports, miRNAs have been shown to bidirectionally regulate angiogenesis in colorectal cancer. Many miRNAs can directly act on VEGF or inhibit angiogenesis through other pathways (HIF-1a, PI3K/AKT, etc.), while some miRNAs, specifically many exosomal miRNAs, are capable of promoting CRC angiogenesis. Understanding the mechanism of action of miRNAs in angiogenesis is of great significance for finding new targets for the treatment of tumour angiogenesis. Deciphering the exact role of specific miRNAs in angiogenesis is a challenge due to the high complexity of their actions. Here, we describe the latest advances in the understanding of miRNAs and their corresponding targets that play a role in CRC angiogenesis and discuss possible miRNA-based therapeutic strategies.

## Introduction

Colorectal cancer (CRC) is the third most common cancer in the world, and is also the second most common cause of cancer-related deaths. Furthermore, in 2018, there were more than 1.8 million new cases of and 881,000 deaths due to CRC [1]. Although most primary colorectal tumours can be surgically removed, the 5-year survival rate of patients with advanced CRC remains low. Metastasis is the leading cause of cancer-related deaths in CRC patients, and it is estimated that more than 50% of patients die of metastasis [2]. Since the concept of angiogenesis was proposed by Maniotis et al. in 1999 [3], a growing number of studies have demonstrated the critical role of abnormal angiogenesis in the invasion and metastasis of CRC. For example, elevated levels of vascular endothelial growth factor-A (VEGF-A) are closely correlated with adverse clinical outcomes in CRC patients [4, 5]. Hence, VEGF-A has been considered a prognostic marker for CRC. In summary, angiogenesis is an important factor that contributes to metastasis in the majority of cancers, including CRC. Undoubtedly, understanding the mechanisms of angiogenesis is necessary to reduce the risk of recurrence and metastasis of CRC.

### Angiogenesis mechanism in CRC

The formation and progression of CRC are inseparable from angiogenesis, and angiogenesis plays an important role in CRC proliferation and metastasis [6]. In general, when a tumour is more than 2 mm in diameter, its growth can no longer be maintained by tissue penetration, resulting in a hypoxic microenvironment of the tumour [7]; therefore, the tumour requires the formation of new blood vessels to provide oxygen and nutrients [8]. After vascular overgrowth, tumour growth and metastasis are promoted by the release of proangiogenic factors, the intensity of which depends on the level of the activation pathway and proangiogenic signals [9]. This process is regulated by a variety of angiogenic factors, such as vascular endothelial growth factor (VEGF), thrombospondin-1 (TSP-1), platelet-derived growth factor (PDGF), transforming growth factor (TGF), endothelial growth factor (EGF), and fibroblast growth factor (FGF) [10–15]. With the continuous research on antiangiogenic therapy that has occurred over the years, targeting angiogenesis has become an important therapeutic strategy for a variety of tumours, including CRC.

As a member of the VEGF family, VEGF-A has been widely recognized as a major participant in tumour angiogenesis [16]. Multiple pathways in a variety of cancers, including CRC, induce VEGF-A expression and promote tumour angiogenesis [17, 18]. Vascular endothelial growth factor receptor 2 (VEGFR2), as a receptor for VEGF, has been shown to be a target for blocking tumour angiogenesis in a number of studies [19]. Bevacizumab is an antagonist of VEGF that significantly inhibits tumour angiogenesis and tumour progression and has been recognized as a first-line treatment for liver metastases in CRC [20, 21].

However, similar traditional antiangiogenic therapies sometimes cause hypoxia and metastasis during treatment, which in turn accelerate tumour growth [22, 23]. The miR-125 family consists of miR-125a, miR-125b-1 and miR-125b-2 [24] and has been shown to be involved in a variety of cancer processes. Members of the miR-125 family appear to have opposite effects in different cancers. For example, miR-125b has been shown to have tumour-suppressing properties in various cancers, including liver cancer [25], oral squamous cell carcinoma (OSCC) [26], and breast cancer [27]. In contrast, miR-125b also is an oncogene in several cancers, including pancreatic cancer [28] and glioblastoma [29]. The different properties of miR-125 family members expressed in various cancers indicate that these miRNAs have highly diverse regulatory functions in cancer progression and that their underlying mechanisms in different cancer environments may differ. Therefore, the molecular mechanism of angiogenesis in CRC must be clarified in a more precise manner. CRC treatment features antiangiogenic agents. Recent studies have found that microRNAs (miRNAs) also play an important regulatory role in the molecular mechanism of tumour angiogenesis, and the identification of key miRNAs has served an important role in the development of more precise CRC targeted therapy strategies.

### Introduction to miRNAs

MiRNAs are small noncoding RNAs of ~ 20–24 nucleotides in length that translationally inhibit or degrade targets by recognizing the 3′ untranslated region (3′-UTR) of mRNAs. As gene expression regulators, miRNAs control ~ 30% of human genes [30–33].

The first miRNA was discovered in nematodes in 1993, and since then, the development of molecular biology techniques has allowed the function of miRNAs to be increasingly and comprehensively studied; as such, the importance of miRNAs has been widely recognized. In recent years, some studies have indicated that miRNAs play a key role in the tumour growth, metastasis and immune response in CRC and that miRNAs can act as oncogenes or tumour suppressors. MiR-196a-5p is a proto-oncogene that regulates CRC epithelial–mesenchymal transition (EMT) by binding to IκBα and promoting CRC proliferation, metastasis and invasion [34]. MiR-500a-5p was confirmed to be a tumour suppressor that was significantly downregulated in CRC, inhibited CRC tumour growth and metastasis by regulating HDAC2 and was negatively regulated by YY1 [35]. Based on recent advances in miRNA-based therapies [36, 37], miRNAs have become an effective treatment and prognostic indicator for CRC. That is, serum miRNAs have been shown to be predictors of CRC tumour recurrence and treatment outcomes [38].

The biogenesis and function of miRNAs are constantly being researched, the understanding of these factors is constantly being improved, and the effect of miRNA regulation on tumour angiogenesis is becoming increasingly clear. MiRNAs regulate angiogenesis during normal physiological processes, such as wound healing, and the aberrant expression of miRNAs can promote and inhibit angiogenesis during tumour progression [9, 39, 40]. Therefore, identifying key miRNAs involved in vascular angiogenesis will play an important role in the development of better therapeutic strategies. In this review, we summarized the exact targets of miRNA that act on CRC angiogenesis in recent years, and explored possible methods for targeted treatment of CRC angiogenesis.

### MiRNAs that inhibit angiogenesis (Fig. 1)

The functions of miRNAs are diverse and affect tumorigenesis, invasion, and metastasis. A series of recent studies have reported the mechanisms by which many miRNAs inhibit angiogenesis in CRC. These reports provide new targets for treatment strategies targeting angiogenesis in CRC (Table 1). VEGFA has been shown to be a good therapeutic target in antiangiogenic strategies [41]. However, the long-term use of VEGF-related drugs often has various side effects. For example, the long-term use of the VEGFA monoclonal antibody bevacizumab induces osteonecrosis [42]. Hence, it is important to search for better targets for angiogenesis therapy and eliminate side effects.

**Fig. 1:**
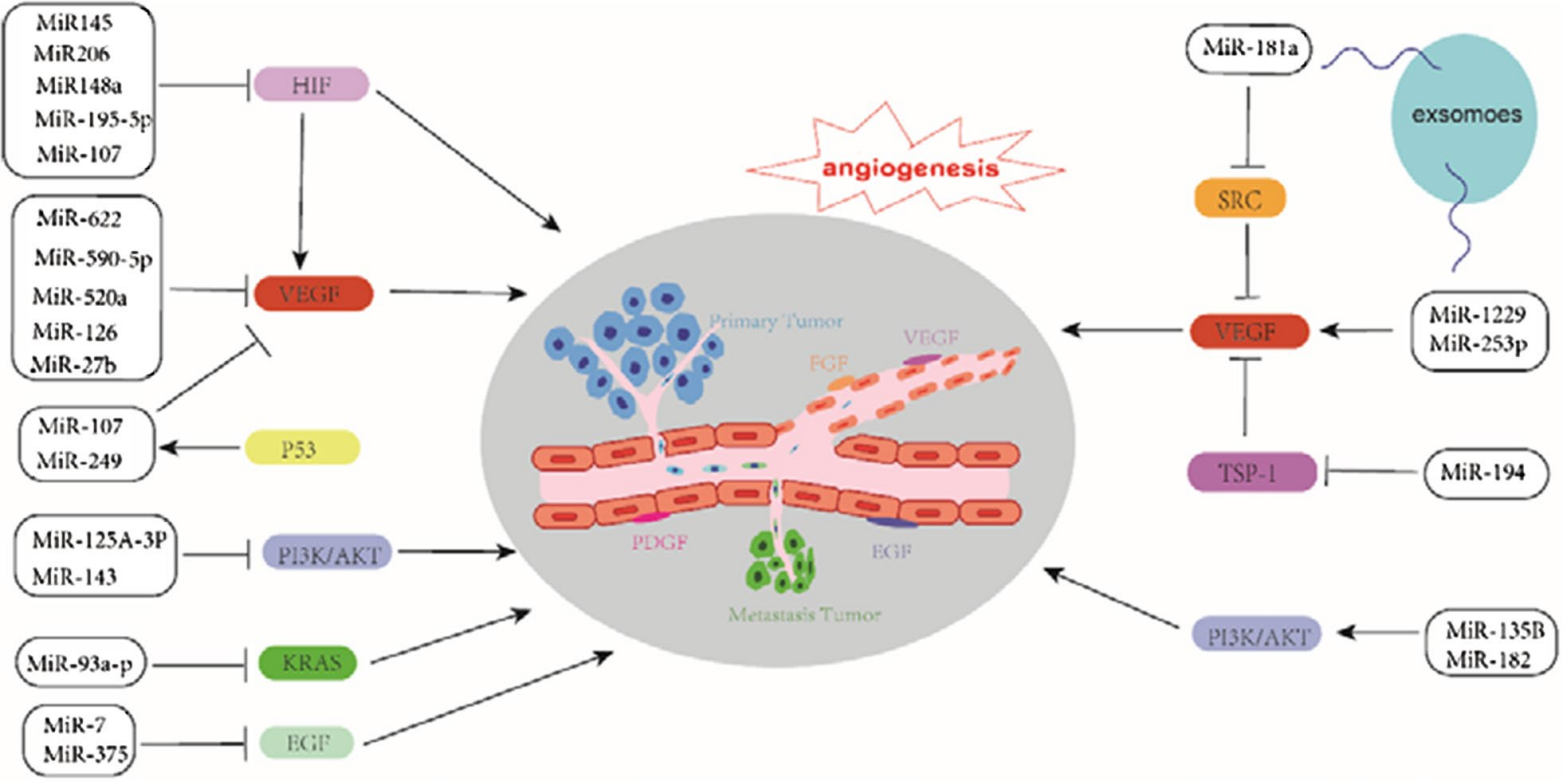
MiRNAs that regulates angiogenesis in colorectal cancer

**Table 1:** miRNAs that inhibit angiogenesis in CRC

MiRNA	Target	References
MiR-107	HIF-1β	[74]
MiR-1249	VEGFA/HMGA2	[76]
MiR-125a-3p	PI3K/AKT	[57]
MiR-126	VEGF	[53]
MIR-1299	STAT3	[81]
MiR-143	PI3K/AKT/HIF-1α/VEGF	[61]
MiR-145	p70S6K1/HIF-1α/VEGF	[69]
MiR-148a	pERK/HIF-1α	[72]
MiR-15-16	c-Myc/Max/HIF-2α/FGF2	[105]
MiR-181a-5p	MMP-14	[109]
MiR-19a	KRAS	[97]
MiR-193a-3P	PLAU	[93]
MiR-206	Met/ERK/Elk-1/HIF-1α/VEGF-A	[71]
MiR-216b	HMGB1	[79]
MiR-27b	VEGFC	[54]
MiR-33a	ST8SIA1	[64]
MiR-375	CTGF/EGFR	[87]
MiR-520a	VEGFA	[51]
MiR-590-5p	VEGFA	[45]
MiR-622	CXCR4-VEGFA	[42]
MiR-6868-5p	FOXM1/IL-8	[101]
MiR-7	EGFR/ERK	[84]
MiR-885-3p	BMP/Smad/Id1	[104]

### MiRNAs targeting VEGF inhibit CRC angiogenesis

Among miRNAs, miR-622 is an miRNA involved in various cancers, such as ovarian cancer, liver cancer, and gastric cancer [43–45], and it has been recently reported to regulate the angiogenesis of CRC by inhibiting CXCR4-VEGFA [46]. VEGF-A is an important receptor for VEGF, is involved in angiogenesis, and triggers the germination of prevascular endothelial cells to induce new vasculature formation. Similarly, miR-590-5p also inhibits the angiogenesis of CRC by affecting VEGF-A [47]. It has been reported that miR-590-5p acts as an oncogene in cervical cancer and a tumour suppressor in renal cancer [48, 49]; miR-590-5p is downregulated in normal tissues compared with CRC tissues, particularly compared with nonmetastatic CRC tissues. MiR-590-5p has been shown to inhibit tumour angiogenesis mainly by inhibiting NF-90/VEGF-A, thereby reducing the enhanced migration ability of CRC cells [47]. MiR-520 was first discovered to act as a tumour suppressor in breast cancer, in which it targets the NF-κB and TBF-β pathways [50], and its family members have also been found to be downregulated in CRC. Furthermore, miR-520a can inhibit the proliferation of oesophageal squamous cell carcinoma by downregulating cysteine-rich C-terminal 1 (CRCT1) [51], and miR-520a-3p can accelerate apoptosis and inhibit cell migration by targeting epidermal growth factor receptor (EGFR) [52]. In addition, miR-520a acts as a direct target of VEGFA, while ATAD2 can inhibit VEGFA secretion by increasing the expression of miR-520a, thereby reducing angiogenesis in CRC [53]. MiR-126 is dysregulated in a variety of cancers, is highly expressed in endothelial cells, and is an angiogenesis inhibitor [54]. Furthermore, miR-126 is usually epigenetically silenced in CRC, and the recovery of miR-126 directly inhibits VEGF expression and reduces angiogenesis, invasion and migration in CRC [55]. MiR-27b inhibits the angiogenesis of CRC by targeting VEGF-C and downregulating DNA hypermethylation, thereby inhibiting the growth of CRC tumours [56]. MiR-150-5p acts as a tumour suppressor in CRC, in which it inactivates Akt/mTOR signalling through direct inhibition of VEGF-A [57]. MiR-125 inhibits VEGF expression by targeting the 3′ untranslated region of VEGF mRNA, thereby promoting apoptosis in RKO CRC cells [58]. The central role of VEGF in the pathogenesis of angiogenesis has also become evident. Instructions of the molecular mechanisms of VEGF and the transformative development of multiple therapeutic pathways targeting VEGF directly or indirectly is a powerful case study of how fundamental research can guide clinical. There are many ways that miRNA targets VEGF to inhibit angiogenesis, which also provides a new theoretical basis for us to search for new targeted drugs.

### MiRNA inhibits CRC angiogenesis via PIK3/AKT

The fucosyltransferase (FUT) family is involved in a variety of cancers, including CRC. In recent reports, miR-125a-3p was shown to be negatively correlated with the expression of FUT5 and FUT6. FUT5 and FUT6 can be used as direct targets of miR-125a-3p, and miR-125a-3p/FUT5-FUT6 attenuates angiogenesis in CRC cells and inhibits tumour growth by affecting the PI3K/AKT signalling pathway [59]. PI3K/AKT signalling plays an important role in the development of tumours, which may cause tumour growth and angiogenesis, and these functions are abnormally activated in a variety of cancers [60, 61]. Several strong inhibitors of tumour angiogenesis targeting the PI3K/AKT pathway have been developed [62]. However, the PI3K/AKT pathway is involved in the biological functions of various normal cells, and thus, the cellular process that depend on the PI3K/AKT pathway can be affected by such treatments. Hence, there is a need to reduce the side effects of related drugs. MiR-143 is a tumour suppressor that is downregulated in CRC and inhibits tumour angiogenesis via the PI3K/AKT/HIF-1α/VEGF pathway [63]. MiR-143 inactivates AKT and inhibits HIF-1α, and VEGF inhibits tumour angiogenesis. Similarly, insulin-like growth factor-I receptor (IGF-IR) had been identified as a direct target of miR-143. MiR-143 can reduce the resistance to oxaliplatin by binding to IGF-IR. Treatment of CRC with different agents has provided new information [63]. MiRNA inhibits the angiogenesis of CRC through the PI3K/AKT pathway, and in recent reports, miR-182 and miR-135b were also shown to promote CRC invasion and angiogenesis via the PI3K/AKT pathway [64]. The expression of miR-182 and miR-135b in tumour tissues and cells is higher than that in normal tissues, and the angiogenesis of CRC is promoted by direct targeting of ST6GALNAC2 to activate the PI3K/AKT pathway. Both ST8SIA1 and ST6GALNAC2 belong to the sialyltransferase (ST) family of enzymes, which promote tumour growth and metastasis [65]. In CRC, miR-33a inhibits tumour angiogenesis and metastasis by regulating ST8SIA1, and the overexpression of miR-33a can inhibit CRC cell resistance [66]. The PI3K/AKT pathway modulates the expression of many angiogenic factors such as VEGF nitric oxide and angiopoietins. Numerous inhibitors targeting the PI3K/AKT pathway have been developed, and these drugs reduce VEGF secretion and angiogenesis. However, their effect on the tumor's vascular system can be difficult to predict. The discovery of miRNA targeting PI3K/ Akt may provide new ideas for this purpose. Activation of the PI3K/ Akt pathway in tumor cells also increases VEGF secretion through hypoxia-inducible factor 1α (HIF-1α) dependent and independent mechanisms.

### MiRNAs that inhibit angiogenesis via HIF-1α

HIF-1α is one of the central molecules that mediate the development of cancer and is a key regulator of VEGF. HIF-1α is involved in tumour angiogenesis in a variety of pathways [17, 67, 68] and is one of the most promising targets for tumour angiogenesis. Various miRNAs are also involved in the regulation of HIF-1α [69]. HIF-1α can regulate miRNAs, and miRNAs can also act on HIF-1α. This bidirectional regulation plays a very important role in tumour progression. Therefore, the mechanism by which miRNAs affect HIF-1α is modulated and is necessary. MiR-145 is downregulated in the early stage of intestinal cancer and is a tumour suppressor [70]. In CRC, miR-145 can inhibit HIF-1α and VEGF by targeting p70S6K1, thereby reducing the angiogenic ability of CRC [71]. MiR-206 is a tumour suppressor, and the downregulation of miR-206 promotes angiogenesis in breast cancer [72]. In CRC, miR-206 acts on the Met/ERK/Elk-1/HIF-1α/VEGF-A pathway to inhibit tumour angiogenesis, and CCL19 can drive this process by promoting miR-206 expression [73]. MiR-148a downregulates VEGF via the pERK/HIF-1α pathway, inhibits tumour angiogenesis and reduces the risk of early recurrence in CRC patients [74]. MiR-195-5p is a multifunctional miRNA that acts as a tumour suppressor in CRC and has not only multiple targets that inhibit CRC cell migration by regulating EMT but also multiple targets that affect blood vessels, such as HIF-1α and VEGF. To downregulate production factors, miR-195-5p has an inhibitory effect on invasion and angiogenic mediators in invasive CRC cells [75].

MiR-107 is a tumour suppressor expressed in human colon cancer specimens and is regulated by P53, which reduces hypoxia signalling by inhibiting HIF-1β expression and reduces tumour angiogenic capacity [76]. P53 is a tumour suppressor. However, due to its susceptibility to mutation, P53 mutation or loss is considered to be a critical step in tumour progression and often suggests poor prognosis of tumours [77]. In a recent report, P53-induced miR-1249 expression was decreased in CRC tissues and cell lines and inhibited CRC metastasis and angiogenesis by affecting VEGFA and HMGA2 both in vivo and in vitro [78]. HMGA2 is a member of the HMGB family, is expressed in a variety of cancers, and is associated with immunopositivity and tumour aggressiveness. Hence, HMGA2 is used as a tumour marker [79]. HMGA1 is another member of the HMGA family and plays a key role in CRC, and HMGA1 overexpression is associated with a lower overall survival rate in patients with CRC [80]. MiR-216b inhibits the proliferation, invasion and angiogenesis of CRC by directly targeting HMGB1. Furthermore, downregulation of miR-216b promotes the progression of CRC by affecting JAK2/STAT3 signalling [81]. As a very important member of the STAT family, STAT3 has been shown to be involved in cancer cell proliferation, metastasis, and angiogenesis in multiple reports, and STAT3 regulates tumour angiogenesis by regulating VEGF and HIF-1α [82]. MiR-1299 promotes the apoptosis of CRC cells by inhibiting the STAT3 pathway and inhibiting CRC. Furthermore, miR-1299 may be a related regulator of angiogenesis. However, further studies are needed [83].

### MiRNAs targeting EGFR to regulate CRC angiogenesis

EGFR is overexpressed in a variety of tumours. Furthermore, it is a carcinogenic factor. EGFR regulates cell proliferation by activating extracellular regulatory protein kinase (ERK) [84]. In addition, EGFR also plays an important role in the vascular growth of CRC, and its targeted inhibitors also play an important role in the treatment of cancer [85]. The expression of EGFR can be regulated by microRNA: miR-7 inhibits the angiogenesis of CRC by downregulating ERK signalling through EGFR. The overexpression of miR-7 downregulates EGFR, ERK1/2 and VEGF and upregulates TSP-1 [86]. EGFR is involved in a variety of cellular responses, such as cell proliferation, differentiation and migration [87, 88]. EGFR binds to CTGF to phosphorylate and activate downstream signalling. MiR-375 inhibits CRC tumour growth, migration and angiogenesis by targeting the CTGF/EGFR-induced downregulation of the PIK3CA-AKT and BRAF-ERK1/2 cascades, thereby acting as a tumour suppressor. In addition, miR-375 and cetuximab also work together to induce an anticancer effect [89]. The results of miR-375 overexpression and cetuximab treatment experiments revealed synergistic enhancement of apoptosis and necrosis of colon cells.

Recent reports have revealed that miR-193a-3P has both cancer-promoting and tumour-suppressing effects, which may be correlated with its environment. However, most studies have reported that miR-193a-3P can inhibit tumour invasion [90–92], and only a few reports have indicated that miR-193a-3P can promote the progression of oesophageal squamous cell carcinoma as a cancer-promoting gene [93]. However, miR-193a-3P is expressed at low levels in CRC tissues and is associated with the prognosis of patients with CRC [94]. In a recent report, miR-193a-3P inhibited the proliferation, migration and angiogenesis of CRC by targeting plasminogen activator urokinase (PLAU) [95]. PLAU is a highly expressed urokinase plasminogen activator (uPA), and its high expression often indicates a poor prognosis. PLAU accelerates tumour metastasis by affecting the ECM and basement membrane, accelerating cell migration and angiogenesis [96]. In ovarian cancer, uPA regulates the AKT/mTOR/MMP-2/Laminin5γ2 signalling pathway to promote angiogenesis [97]. However, in CRC, miR-193a-3P can inhibit tumour angiogenesis by downregulating PLAU [94]. Since the exact downstream pathway remains unclear, further research is still needed. Recent reports have indicated that miR-193a-3P can inhibit tumour progression by targeting KRas in lung cancer.

### KRAS and miRNAs affect angiogenesis

The KRAS (k-ras, p21) gene plays an important role in regulating tumour growth and angiogenesis. Activation of KRAS mutations induces CRC cell growth, invasion, and metastasis; and thus is considered a critical step in the progression of CRC [98]. KRAS gene detection is the most direct and effective method to understand the status of oncogenes in patients with colorectal cancer. Through detection of KRAS, we can understand the status of oncogenes, so as to screen out targeted drugs against EGFR. Recently, highly expressed miR-19a was shown to downregulate KRAS to reduce angiogenesis, and specifically angiogenesis in CRC, and this effect was restored after the re-expression of KRAS, indicating that miR-19a can directly regulate KRAS and reduce the angiogenesis of CRC [99]. Other studies have shown that miR-19a is negatively correlated with TF expression in patients with early colon cancer, and can inhibit TF expression in vitro and inhibit the migration and invasion of CRC [100]. Interestingly, in other studies, miR-19a showed a different effect. These studies have shown that miR-19a promotes the proliferation and migration of colorectal cancer [101–103], and that miR-19a is also associated with lymphatic metastasis and mediates TNF-α-induced epithelial mesenchymal transformation in colorectal cancer [104]. The differences in the above research results, may be related to the different cell lines selected, or may be caused by an undiscovered mechanism, just like miRNAs play the opposite role in different cancers, such as miR-125b in breast cancer both promote cancer [105] and inhibiting cancer [106]. Therefore, it is particularly necessary to clarify the specific mechanism of miRNA's regulation of angiogenesis, which is of great significance for the application of miRNA to target angiogenesis in the treatment of tumors.

### FOXM1 and miRNAs in angiogenesis

Forkhead box M1 (FOXM1) is a member of the FOX superfamily. It is overexpressed in a variety of cancers, including CRC [107], in which it promotes EMT, angiogenesis, cell proliferation, stem cell self-renewal, etc. As an activator of tumour metastasis, it exhibits a wide variety of cancer-promoting properties. FOXM1 is a major regulator of CRC and can be used as an indicator of poor prognosis [108, 109]. Recent studies have shown that miR-6868-5p is able to inhibit tumour angiogenesis by inhibiting the FOXM1-IL-8 axis. In turn, FOXM1 also downregulates miR-6868-5p by stimulating EZH2-mediated transcription [110]. In general, the expression of miR-6868-5p is downregulated in CRC, and downregulation of miR-6868-5p inhibits tumour angiogenesis through inhibition of FOXM1; FOXM1 can also inhibit miR-6868-5p expression through promoter histone methylation. The pro-angiogenic factor IL-8 has been identified as a transcriptional target of FOXM1, and it was revealed that miR-6868-5p reduces angiogenesis and IL-8 expression by inhibiting FOXM1 expression. FOXM1 can promote the expression of EZH2 and inhibit the transcription of miR-6868-5p by enhancing the level of H3K27me3 at the miR-6868 promoter [110]. These findings provide a new perspective on the mechanism of CRC angiogenesis.

### BMPs affect angiogenesis with miRNAs

Bone morphogenetic proteins (BMPs) are also involved in angiogenesis; they trigger signalling through BMP/Smad to affect angiogenesis, and ID1 is their immediate downstream effector. The DNA-binding protein inhibitor ID-1 (ld1) is overexpressed in tumours and inhibits tumour angiogenesis in mice. As one of the most potent angiogenic factors, ID1 can serve as a direct antiangiogenic target [111, 112]. In a recent study, it was reported that miR-885-3p could inhibit the angiogenesis of CRC by regulating BMPR1A to disrupt the BMP/Smad/Id1 signalling pathway, thereby inhibiting the growth of CRC cells [113].

### Other miRNAs that regulate angiogenesis

MiR-15-16 is a tumour suppressor that has been shown to promote apoptosis and inhibit cell proliferation in various independent studies. In addition, recent research reported that c-Myc/Max, HIF-2α, miR-15-16 and FGF2 signalling regulation can modulate CRC angiogenesis under hypoxic conditions. Under hypoxic conditions, HIF-2α-induced c-Myc/Max heterodimer stability is much stronger than HIF-1α-induced c-Myc degradation, resulting in the inhibition of miR-15-16 under hypoxic conditions. The enhanced expression of FGF2 promotes tumour angiogenesis and metastasis [114].

MiR-181a-5p belongs to the miR-181 family, which includes miR-181a, miR-181b, miR-181c and miR-181d. Recent reports have indicated that such miRNAs play an important role in tumour transformation. Furthermore, some studies have shown that miR-181 members promote the formation of tumours [115]. However, miR-181a-5p is specific tumour marker, and it has been reported to promote the progression of cervical cancer [116]. On the other hand, miR-181a-5p has been shown to inhibit tumour growth in liver cancer [117]. In breast cancer and colon cancer, there is evidence that miR-181a-5p can inhibit the invasion and migration of cancer cells and angiogenesis by inhibiting the expression of MMP-14 [118]. These controversial findings indicate the complexity of miRNA functions and that the functions of miRNAs in different types of tumours may significantly differ. However, various studies use tumour tissues, which include a variety of normal cells and tumour cells, and this practice may lead to incorrect conclusions about the expression levels of specific miRNAs. As such, further research is needed.

### MiRNAs that promote angiogenesis (Table 2)

MiR-181a also plays a role in a variety of tumours. For example, in chondrosarcoma, miR-181a increases VEGF to promote tumour growth [119]. Unlike miR-181a-5p, which was shown to inhibit angiogenesis in CRC, miR-181a was shown to inhibit angiogenesis in CRC in a recent study. MIR-181a activates SRC by inhibiting SRCIN1, which ultimately leads to increased secretion of VEGF and promotes angiogenesis in CRC. This study demonstrated that the miR-181a-SRCIN1-SRC-VEGF cascade plays an important role in the regulation of tumour angiogenesis and that blocking this pathway can significantly reduce tumour angiogenesis [120]. MiR-194 reduces platelet-reactive protein-1 (TSP-1), reducing its damage to endothelial cells and effects on VEGF and promoting angiogenesis in colon cancer [121].

**Table 2:** MiRNAs that promote angiogenesis in CRC

MiRNA	Target	References
MiR-1229	HIPK2	[117]
MiR-1246	PML/Smad 1/5/8	[119]
MiR-135b	PI3K/AKT	[62]
MiR-181a	SRCIN1	[111]
MiR-182	PI3K/AKT	[62]
MiR-194	TSP-1	[112]
MiR-25-3p	KLF2/KLF4/VEGF	[118]

Exosomes are extracellular small vesicles that contain lipids, proteins and various nucleic acids, including RNA, DNA and miRNA. Most cells secrete exosomes under normal and pathological conditions, while tumour exosomes are derived from outside the tumour. The body includes many species of miRNA [122, 123]. Exosomes are one of the cancer-derived factors that cause distant organ metastasis and promote tumour angiogenesis and metastasis [124, 125]. However, determining how tumour-derived exosomes specifically regulate the tumour microenvironment before metastasis by inducing angiogenesis requires more research. In a recent study, it was shown that exomiR-1229 can promote CRC angiogenesis by directly regulating HIPK2. HIPK2 can inhibit several angiogenic genes, including MMP10 and VEGF, to inhibit angiogenesis, while exomi-1229 promotes CRC tumour angiogenesis by inhibiting HIPK2 and inhibiting P-AKT and VEGFA in CRC [126]. CRC-secreted miR-25-3p can inhibit the activity of the VEGFR2 promoter and disrupt the integrity of the endothelial barrier by regulating KLF2 and KLF4, resulting in increased vascular permeability and angiogenesis and thereby promoting CRC metastasis. In addition, blocking its secretion can reduce the angiogenesis and metastasis of CRC [127]. It has been well demonstrated that miR-25-3p can be used as a therapeutic target to disrupt CRC angiogenesis and metastasis. MiR-1246 secreted by colorectal cancer exosomes directly regulates promyelocytic leukaemia (PML) mRNA, which inactivates Smad2/3 signalling and activates Smad-1/5/8 signalling, leading to endothelial cell growth and tumour angiogenesis [128].

### Clinical studies based on miRNAs affecting angiogenesis

In recent years, antiangiogenic strategies have become an important treatment for metastatic colorectal cancer (mCRC), and various antiangiogenic agents have emerged. However, there are still no validated markers for the treatment of angiogenesis. MiRNAs have been shown to regulate angiogenesis in a variety of cancers. Comprehensive miRNA-based therapies may have better efficacy and fewer adverse effects than conventional chemotherapy. Bevacizumab, which inhibits VEGF-A, has been used in a variety of therapeutic regimens [129]. Furthermore, recent studies have shown that miR-20b-5p, miR-29b-3p and miR-155-5p can be used to monitor the response to bevacizumab, and high expression levels of these miRNAs often suggest good prognosis in mCRC patients treated with bevacizumab. Measuring the expression of these miRNAs before treatment helps to identify patients who will likely be resistant to bevacizumab therapy, thereby enabling a better treatment regimen to be administered [130]. Hanen et al. [131, 132] demonstrated that miR-126 has predictive value for the first-line capecitabine and oxaliplatin treatment in mCRC. Highly expressed miR-126 is often associated with anti-VEGFA chemotherapy response in mCRC, in which high expression of miR-126 is related to good prognosis.

## Conclusion

Angiogenesis plays an important role from the early stage of colon cancer to the late phase of metastasis. There is ample evidence that angiogenesis is a complex process that involves multiple factors and processes. MiRNAs have a large impact on such factors and processes, and they are potential targets for chemoprevention and chemotherapy. Studies have shown that miRNAs, as a new therapeutic target for tumours, can be successfully regulated through a series of techniques. For example, miRNA antagonists and mimetics may be important new classes of drugs that regulate angiogenesis in colon cancer. A good understanding of the role of miRNAs in the regulation of angiogenesis can help to fully understand the function of miRNAs and their targets and can provide more pathways to target with miRNA-based therapeutic applications. However, there are relatively few clinical studies on the role of miRNAs in CRC angiogenesis. Despite some challenges, numerous discoveries have been made over the past few years, providing a broad perspective for the use of miRNAs in future targeted therapies.

## Data Availability

Not applicable.

## Abbreviations

CRC: Colorectal cancer
VEGF: Vascular endothelial growth factor
TSP-1: Thrombospondin-1
PDGF: Platelet-derived growth factor
TGF: Transforming growth factor
EGF: Endothelial growth factor
FGF: Fibroblast growth factor
OSCC: Oral squamous cell carcinoma
PI3K: Phosphatidylinositol 3-kinase
AKT: Protein kinase B
EMT: Epithelial–mesenchymal transition
HDAC2: Histone deacetylase
CRCT1: Cysteine-rich C-terminal 1
ATAD2: ATPase family AAA domain-containing protein 2
FUT: Fucosyltransferase
IGF-IR: Insulin-like growth factor-I receptor
STs: Sialyltransferases
HMGA2: High‑mobility group AT‑hook 2
PLAU: Plasminogen activator urokinase
uPA: Rrokinase plasminogen activator
FOXM1: Forkhead box M1
BMP: Bone morphogenetic protein
PML: Promyelocytic leukaemia
